# Comparative Study of the Osteogenic Differentiation Potential of Adipose Tissue-Derived Stromal Cells and Dedifferentiated Adipose Cells of the Same Tissue Origin under Pro and Antioxidant Conditions

**DOI:** 10.3390/biomedicines10123071

**Published:** 2022-11-29

**Authors:** Anne Bollmann, Hans Christian Sons, Jennifer Lynn Schiefer, Paul C. Fuchs, Joachim Windolf, Christoph Viktor Suschek

**Affiliations:** 1 Department for Orthopedics and Trauma Surgery, Medical Faculty and University Hospital Duesseldorf, Heinrich-Heine-University Düsseldorf, Moorenstraße 5, 40225 Düsseldorf, Germany; 2 Department of Plastic Surgery, Hand Surgery, Burn Center, Merheim Hospital Cologne, University of Witten/Herdecke, Ostmerheimer Straße 200, 51109 Köln, Germany

**Keywords:** osteogenesis, adipose stem cell, dedifferentiated fat cell, oxidative stress, reactive oxygen species, catalase

## Abstract

Adipose tissue-derived stromal cells (ASCs) play an important role in various therapeutic approaches to bone regeneration. However, such applications become challenging when the obtained cells show a functional disorder, e.g., an impaired osteogenic differentiation potential (ODP). In addition to ASCs, human adipose tissue is also a source for another cell type with therapeutic potential, the dedifferentiated fat cells (DFATs), which can be obtained from mature adipocytes. Here, we for the first time compared the ODPs of each donors ASC and DFAT obtained from the same adipose tissue sample as well as the role of oxidative stress or antioxidative catalase on their osteogenic outcome. Osteogenic potential of ASC and DFAT from nine human donors were compared in vitro. Flow cytometry, staining for calcium accumulation with alizarin red, alkaline phosphatase assay and Western blots were used over an osteogenic induction period of up to 14 days. H_2_O_2_ was used to induce oxidative stress and catalase was used as an antioxidative measure. We have found that ASC and DFAT cultures’ ODPs are nearly identical. If ASCs from an adipose tissue sample showed good or bad ODP, so did the corresponding DFAT cultures. The inter-individual variability of the donor ODPs was immense with a maximum factor of about 20 and correlated neither with the age nor the sex of the donors of the adipose tissue. Oxidative stress in the form of exogenously added H_2_O_2_ led to a significant ODP decrease in both cell types, with this ODP decrease being significantly lower in DFAT cultures than in the corresponding ASC cultures. Regardless of the individual cell culture-specific ODP, however, exogenously applied catalase led to an approx. 2.5-fold increase in osteogenesis in the ASC and DFAT cultures. Catalase appears to be a potent pro-osteogenic factor, at least in vitro. A new finding that points to innovative strategies and therapeutic approaches in bone regeneration. Furthermore, our results show that DFATs behave similarly to ASCs of the same adipose tissue sample with respect to ODPs and could therefore be a very attractive and readily available source of multipotent stem cells in bone regenerative therapies.

## 1. Introduction

Healing critical size bone defects and osteoporotic bones is still a major challenge for both the patient and the attending physician [1]. Either the defect is too large to properly heal on its own or other factors hinder sufficient recovery [2,3]. Mesenchymal stromal stem cells (MSC) like bone marrow derived stromal stem cells (BMSC) can already be used to treat some of these conditions [4,5]. Although bone marrow has long been regarded as the most suitable source for therapeutically relevant MSCs, there is now much more focus on other adult tissue types that are promising sources for potent MSC, such as adipose tissue [6].

The adipose tissue is an efficient source for the isolation of large quantities of adipose tissue-derived stroma cells (ASC). ASCs are a heterogenic cell population consisting of fibroblasts, pericytes, endothelial cells, preadipocytes and hematopoetic cells [7]. Nevertheless, ASCs exhibit all properties of MSC, including the expression of MCS-typical surface markers [8]. Furthermore, a characteristic trait of ASCs is a three lineage differentiation potential to adipocytes, chondrocytes and osteoblasts [9]. In contrast to BMSCs, however, ASCs can be obtained more easily and with fewer side effects and are now much more than just another alternative cell source for regenerative therapy options. However, it should not be left unmentioned that due to the pronounced heterogeneity with regard to the cellular composition, it is much more difficult to predict the therapeutic success than when using a cell therapeutic agent with a defined cell type.

Adipose tissue contains another, but in contrast to ASC, very homologous source of a cell type with a pronounced MSC character. These are dedifferentiated fat cells (DFATs) which can be isolated from the same adipose tissue as ASCs via dedifferentiation of adipocytes by so called ceiling culture [10,11]. The resulting DFATs are a cell type of high uniformity with a fibroblast-like phenotype, ultimately losing their last lipid droplets after passaging. They exhibit the typical MSC-like antigen phenotype and have three lineage differentiation potential [10,11]. 

It is a well-known phenomenon that mesenchymal stromal cells can often have an impaired, disturbed or even absent osteogenic capacity. The physiological or pathophysiological factors underlying this phenomenon are extremely broad. Previous reports suggest that a decline in the number of mesenchymal stem cells or the differentiation potential of stem cell populations may contribute to human aging and age-related diseases [12,13]. In a mouse animal model, it could be shown that bone marrow cells show age-associated differences in osteoblast differentiation [14,15]. However, an age-related decrease in the osteogenic potential of human bone MSCs could also be demonstrated in humans by determining molecular parameters [16], an observation which is based on an age-related change in intrinsic factors [17,18]. Besides age, other factors also seem to influence the osteogenic differentiation potential of human mesenchymal stem cells. Some evidence suggests that obesity decreases the osteogenic differentiation capacity of ASCs. Thus, in different user models, ASC cultures isolated from lean donors showed an improved and much stronger osteogenic differentiation than ASC cultures isolated from obese donors [19]. 

It now seems to be generally accepted that reactive oxygen species and the associated oxidative stress represent the main molecular mechanism behind the age- or obesity-related decrease in the osteogenic potential of mesenchymal stem cells [20]. It is postulated that oxidative stress, which is characterized by an increased presence of reactive oxygen species like superoxide anion (O_2_^−^), hydroxyl radical (•OH), hydrogen peroxide (H_2_O_2_), hypochlorous acid (HOCl), peroxyl or hydroperoxyl radicals (ROO•, HOO^−^), plays a special role as negative factors for osteogenesis [21,22]. An increased production of ROS can be the result of external environmental factors such as smoking [23,24] or UV exposure [25,26], but also incorrectly regulated endogenous processes. Enzymes from the NADPH oxidase family play a role as do complex I and III of the mitochondrial respiratory transport chain [27,28]. 

On the other hand, one can assume that the protective systems of the cell with an antioxidant effect, such as the enzymes MnSOD or catalase that can effectively break down ROS, could possibly also have a positive effect on the osteogenic differentiation potential of oxidatively stressed MSC [20]. MnSOD converts highly reactive O_2_^−.^ to oxygen and less reactive H_2_O_2_. Catalase reduces H_2_O_2_ to water (2 H_2_O_2_ → O_2_ + 2 H_2_O) [29]. Studies in human BMSCs showed a decrease in intracellular H_2_O_2_ and O_2_^−^ levels in osteogenic differentiation as well as an upregulation of antioxidative enzymes catalase and MnSOD [30]. H_2_O_2_ addition decreased alkaline phosphatase (ALP) activity in murine preosteoblastic cells (MC3T3-E1) and BMSC (M2-10B4) cell lines [31], an important osteogenic protein which calcifies osteoblasts extracellular matrix. 

In the present study, we examined in vitro the impact of oxidative stress, especially of hydrogen peroxide, on the osteogenic differentiation capacity of ASC and DFAT cultures obtained from the same human adipose tissue samples. Furthermore, we analyzed the effect of exogenously applied catalase on the osteogenic differentiation potential of osteogenic compromised human ASCs and DFATs.

## 2. Materials and Methods

### 2.1. Materials

If not indicated otherwise, all chemicals were obtained from Sigma (Deisenhofen, Germany), plastic wares were obtained from Greiner (Frickenhausen, Germany), and culture media were purchased from Gibco (Carlsbad, CA, USA).

### 2.2. Source of Adipose Tissue

Adipose tissue either originated from humans undergoing abdominal flap surgery or from liposuctions. In all cases it was tissue from subcutaneous areas. Donors were made anonymous but in most cases gender, age and kind of surgery were documented (see Supplementary Table S1). For most of the osteogenic experiments’ cells of *n* = 9 donors were used. Abdominal flap pieces were transported to the laboratory right after surgery in DMEM at 4 °C. Lipoaspirate was harvested by Coleman technique and transported at 4 °C. Transport time was about 1 h. Tissue was processed immediately after arrival at the laboratory. Samples were used with donors’ consent and in accordance with the guidelines of the ethics committee of the University Hospital Düsseldorf (study number 3634 from 17 July 2011) and in compliance with the Declaration of Helsinki Principles (revision of October 2013).

### 2.3. Processing of Adipose Tissue and Isolation of ASCs and Mature Adipocytes

Adipose tissue was minced under sterile conditions using Petri dish, scalpel and forceps. It was transferred to 50 mL conical tubes adding collagenase I solution in a 1:1 ratio (0.2% type: CLS 255 U/mg). Lipoaspirate was transferred into 50 mL conical tubes and centrifuged at 300 g for 10 min. Two layers were generated. The topmost layer is the layer of interest, which is transferred into new tubes in a 1:1 ratio with collagenase solution. Tissue was digested at 37 °C under gentle shaking for 1 h. Digested tissue was filtered through 100 µM gauze. Filtrate was centrifuged at 300× *g* for 10 min. Three layers were generated. The topmost oil was aspirated and discarded. The middle layer contained the mature adipocytes. They were transferred into new tubes for DFAT culture. The third layer was aspirated except for the cell pellet, which contained the ASC fraction. The cell pellet was washed in NaCl solution (0.9%) for 5 min at 300× *g*. ASCs were seeded into cell culture flasks with medium (see cultivation of ASCs and DFAT) and incubated at 37 °C and 5% CO_2_. On the next day, medium was exchanged to get rid of floating erythrocytes [32].

### 2.4. Dedifferentiation of Mature Adipocytes

DFATs were generated by ceiling culture. Then, 3 mL of mature adipocytes from the isolation process (middle layer) were seeded into T25 cell culture flasks, which were filled with medium (see Culture conditions of ASCs and DFAT cultures) up to the brim. Flasks were turned upside down and incubated at 37 °C for 10 days (Supplementary Figure S2). Floating adipocytes adhered to the tops’ plastic and started dedifferentiation, by losing their contained lipid. After 10 days, flasks were turned to their regular side. After 1 h, in which loose adipocytes and oil could float to the surface, medium was aspirated. 5 mL of fresh medium was added. Cells were then cultured under normal conditions at the bottom of the flask. They developed a fibroblast-like phenotype. After passaging no lipid droplets were observed in DFATs.

### 2.5. Culture Conditions of ASC and DFAT Cultures

ASCS and DFATs were cultured in DMEM with 5% FCS (1 g glucose/500 mL, + glutamine), FGF2 (2 ng/mL) and 1% penicillin/streptomycin at 37 °C and 5% CO_2_. Cells were expanded in T125 cell culture flasks and cryo-conserved in FCS-10% DMSO at −80 °C. Cells were transferred from passage 2 to p3 for the experimental setups. Experiments were performed in 24 well plates, 20,000 cells were seeded per well and experiments started 3 days after seeding. Each well contained 1 mL of medium. To verify equal division of the cells into the wells we used an Eppendorf Dispenser and performed CellTiter-Blue® Assay for cell viability right before switching cells to osteogenic differentiation medium. CellTiter-Blue® Assay (Promega, Mannheim, Germany) confirmed equal amounts of cells per well. Because of the antioxidant properties of catalase and thus its reducing ability of the CellTiter-Blue® dye resazurin, resulting in unreliable results, the assay was not performed in the osteogenic process.

### 2.6. Induction and Quantification of Osteogenic Differentiation

Osteogenic differentiation of ASCs and DFATs was induced by DMEM containing 4.5 g/L glucose, 10% FCS, 1% Penicillin/Streptomycin, 100 nM dexamethasone, 50 µM L-ascorbic-2-phosphate and 10 mM β-glycerophosphate [32] and the degree of mineralization of the extracellular matrix as main parameter of osteogenic differentiated was quantified by Alizarin Red S staining [33] exactly as described previously [34,35].

### 2.7. Induction and Quantification of Chondrogenic Differentiation

Chondrogenic differentiation ASCs and DFATs was induced by maintaining cell cultures in DMEM containing 4.5 g/L glucose, 1% FCS, 1% Penicillin/Streptomycin, 50 nM L-ascorbic-2-phosphate, 1125 µM insulin and 10 ng/mL TGF-β1 [36]. Proteoglycane-containing extracellular matrix as main parameter of ongoing chondrogenesis was stained by alcianblue (1% solution, Merck, Darmstadt, Germany) and extinction at 600 nm of alcianblue extracted by isopropanol/hydrochloric acid served as quantitative parameter of chondrogenic differentiation exactly as described previously [37,38].

### 2.8. Induction and Quantification of Adipogenic Differentiation

Adipogenic differentiation of ASCs and DFATs was achieved by cultivating cell cultures in the first week in DMEM/F12 medium containing 1% Penicillin/Streptomycin, 2 mM L-glutamine, 10 µM dexamethasone, 0.5 mM IBMX, 66 nM insulin, 1 nM trijodthyronine, 10 µg/mL transferrine, 0.1 µg/mL rosiglitazone and in the second and third week containing 1% Penicillin/Streptomycin, 2 mM L-glutamine, 1 µM dexamethasone, 66 nM insulin, 1 nM trijodthyronine and 10 µg/mL transferrine exactly as described earlier [39,40,41]. Medium was changed every 3–4 days for 21 days total. At day 21 cells lipid vesicles were stained with Nile red and fluorescence (excitation at 485 nm and emission at 535 nm) was measured with a Multilabel counter (Victor3, PerkinEllmer). Each wells Nile red values were normalized to CelltiterBlue Fluorescence (excitation: 540 nm/emission: 590 nm). 

### 2.9. Alkaline Phosphatase (ALP) Assay

The colorimetric Alkaline Phosphatase Assay Kitt (Abcam, Cambridge, UK) was used to quantify alkaline phosphatase activity of resting and osteogenically activated ASC and DFAT cultures exactly as recommended in the manufacturer’s instructions for use. 

### 2.10. Cell Viability Assay

Cell proliferation and cell amount was measured by evaluating the cell metabolism/viability via CellTiterBlue Assay (Promega, Manheim, Germany) following manufactures instructions. 

### 2.11. Use of H_2_O_2_ and Antioxidants

The influence of oxidative stress onto the osteogenic differentiation potential of ASCs and DFATs was simulated by complementing the osteogenic differentiation medium with H_2_O_2_ (50–400 µM). The situation of an antioxidant effect was simulated by adding catalase (250 U/mL). 

### 2.12. FACS Analysis of Stroma Cell Phenotype

In order to characterize the cell surface antigen phenotype of isolated ASC and their corresponding DFAT cultures, adherently growing cells were detached by 0.5% trypsin and 0.02% EDTA, washed (centrifuged at 200× *g* for 5 min), and stained for 30 min on ice with fluorescent dye conjugated MSC-relevant antibodies against CD13, CD14, CD19, CD26, CD29, CD31, CD34, CD44, CD45, CD73, CD90, CD105, CD146, HLA-DR, BMPR1A, BMPR1B, TGFβR1, TGFβR2, and PPARγ for 30 min [42,43]. Labeled cells were analyzed using a FACSCalibur (BD biosciences, Heidelberg, Germany). In the case of PPARγ detection cells were first fixed with CellFix, washed, permeabilized (cellwash, 0.2% Triton) and incubated with α-PPARγ antibody (H-100, Santa Cruz BT, Heidelberg, Germany). After washing, cells were incubated with secondary antibody (α-rabbit IgG-NL557, R&D Systems, Wiesbaden, Germany) washed once more and then analyzed. There are different numbers of *n*. The *n* = 11 includes 3 additional donors data of previous experiments. CD146, BMPs and TGFs are *n* = 6, CD14 and 34 are *n* = 8, PPARy is *n* = 3. 

### 2.13. Western Blotting

Western Blot analysis of protein expression was perform exactly as described by us recently [44]. 20 µg of protein was used. As primary antibodies we used monoclonal mouse-anti MnSOD, catalase (Cell Signaling, Frankfurt am Main, Germany) and GAPDH (Novus, Cambridge, Great Britain) antibodies (1:1000). After incubation, membranes were washed 3 × 5 min in TBS-T (0.1% Tween 20) and incubated with secondary antibody in 5 % TBS-T (1:1000) for one hour at room temperature. As secondary antibodies we used HRP-conjugated polyclonal goat-anti-mouse antibodies (Dako, Carpinteria, CA, USA). After washing (3 × 5 min in TBS-T), membranes were incubated in enhanced luminescence reagents (Thermo Fisher Scientific). Blots were analyzed with ImageLab software (Bio-Rad). Specific protein signals were normalized to the respective GAPDH signals or to the signal of total proteins detected on the respective lanes of the gel prior blotting.

### 2.14. Statistical Analysis

The statistical evaluation was carried out using the Graph Pad Prism 5 and 8 software. We used the paired two-tailed Student’s *t*-test or ANOVA followed by a post hoc multiple comparison test (Tukey). With a *p*-value < 0.05, the statistical differences were classified as statistically significant.

## 3. Results

### 3.1. Phenotype and Multilineage Differentiation of ASC and DFAT Cultures Obtained from the Same Adipose Tissue

**Figure 1:**
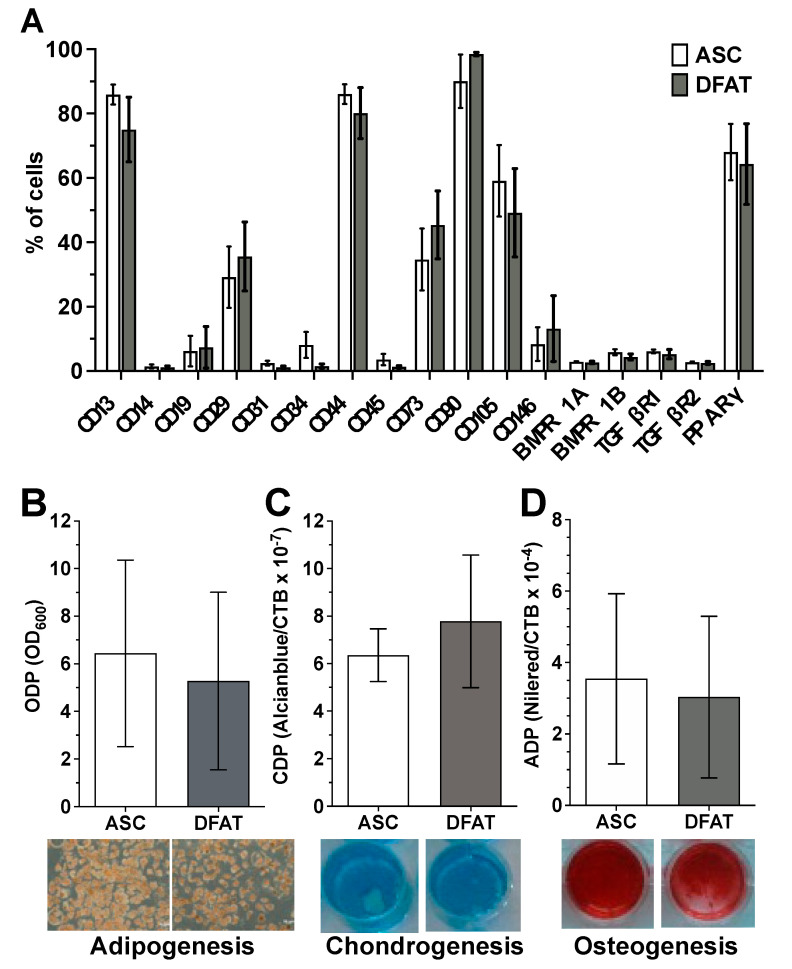
Mesenchymal stem cell character of the established ASC and DFAT cultures obtained from the same adipose tissue sample. (**A**) Flow cytometry-based characterization of the surface antigen phenotype of ASCs and their corresponding DFATs in passage 2. CD146 and BMPs and TGFs are *n* = 6, CD14 and 34 are *n* = 8, all remaining CDs are *n* = 11. PPARy *n* = 3. (**B**–**D**), Evidence of plastic adherence and multilineage differentiation potential of ASC and DFAT cultures obtained from the same adipose tissue: adipogenic (**B**), chondrogenic (**C**), osteogenic (**D**) differentiation potential, scale bar 200µM. Adipo- (at day 21, *n* = 6), Chondrogenesis (at day 24, *n* = 6), Osteogenesis *n* = 15 (at day 14, including 6 additional donors’ data), paired *t*-test and mean + SD.

Flow cytometry-based characterization of the surface antigen phenotype of ASCs and their corresponding DFATs in passage 2 did not reveal any significant differences in the expression pattern of the examined surface proteins between both cell type (Figure 1A and Supplementary Figure S3). Both cell types show no or only a negligibly low expression of the hematopoietic markers CD14, CD19 and CD45, the endothelial markers CD31 and CD34 as well as the perivascular cell marker CD146. In contrast ASCs and DFATs are positive for CD13, CD29, CD44, CD73, CD90 and CD105 which are typical positive MSC markers. In addition, both cell types are nearly negative for osteogenic surface markers BMPR1A/B and TGFβR1/2 and express intracellular adipogenic transcription factor PPARγ (Figure 1A). 

ASC and DFAT cultures also showed other properties that are characteristic of mesenchymal stem cells. They showed pronounced adherence to the plastic cell culture plates used and a strong, significant multilineage, adipogenic (Figure 1B), chondrogenic (Figure 1C) and osteogenic (Figure 1D) differentiation potential. 

### 3.2. Comparison of the Osteogenic Differentiation Potential of ASC and DFAT Cultures Obtained from the Same Adipose Tissue

**Figure 2:**
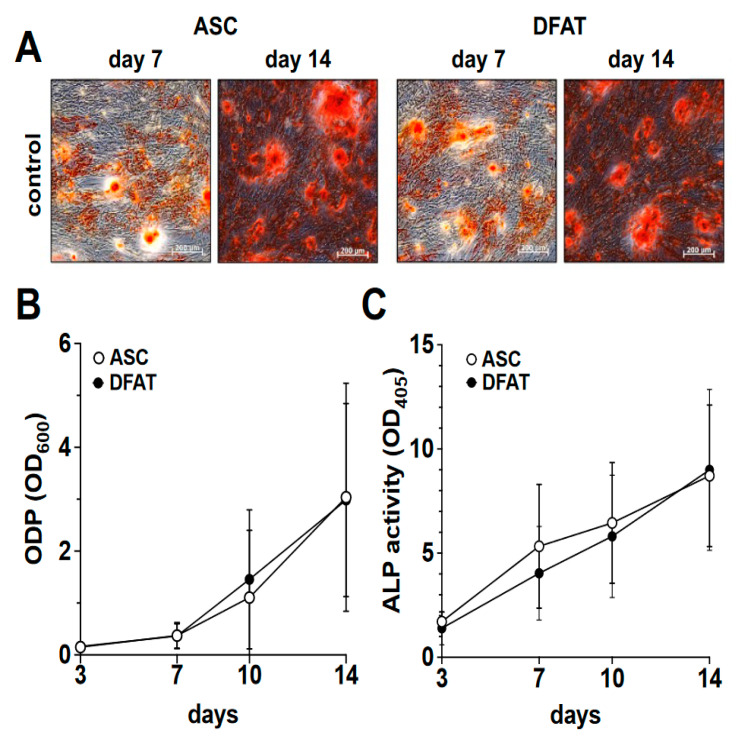
Osteogenic differentiation potential of ASC and DFAT cultures obtained from the same adipose tissue sample. (**A**) Alizarin Red stain of osteogenically activated ASC and DFAT cultures at day 7 and day 14 of differentiation, scale bars 200 µM. (**B**) Quantitative evaluation of the osteogenic differentiation potential (ODP) of osteogenically activated ASC (white circles) and DFAT (black circles) cultures. The values show the mean ± standard deviation of values (*n* = 9) of the optical density of the Alizarin Red redissolution at 600 nm (OD_600_) of the cultures and days of differentiation indicated. (**C**) Quantitative evaluation of alkaline phosphatase (ALP) activity of osteogenically activated ASC (white circles) and DFAT (black circles) cultures. The values show the mean ± standard deviation of values (*n* = 9) of the optical density of the ALP-assay-specific alkaline phosphatase product 405 nm (OD_405_) of the cultures and days of differentiation indicated.

In order to compare the osteogenic differentiation of ASC and DFAT cultures of the same tissue origin, we osteogenically differentiated the corresponding cultures over a period of 14 days. On days 3, 7, 10 and 14 of differentiation, we quantified the degree of calcification of the extracellular matrix as a measure of osteogenic differentiation using alizarin red and the activity of alkaline phosphatase (Figure 2) using the corresponding activity assay.

As we show in Figure 2A,B, a steadily increasing degree of osteogenic differentiation of the ASC and DFAT cultures could be observed in the examined time interval of 14 days. At no time was a statistically significant difference in the osteogenic differentiation potentials of the two cell types found. With regard to the activity of alkaline phosphatase, we were able to observe an almost identical course in the corresponding ASC and DFAT cultures over this period (Figure 2C).

**Figure 3:**
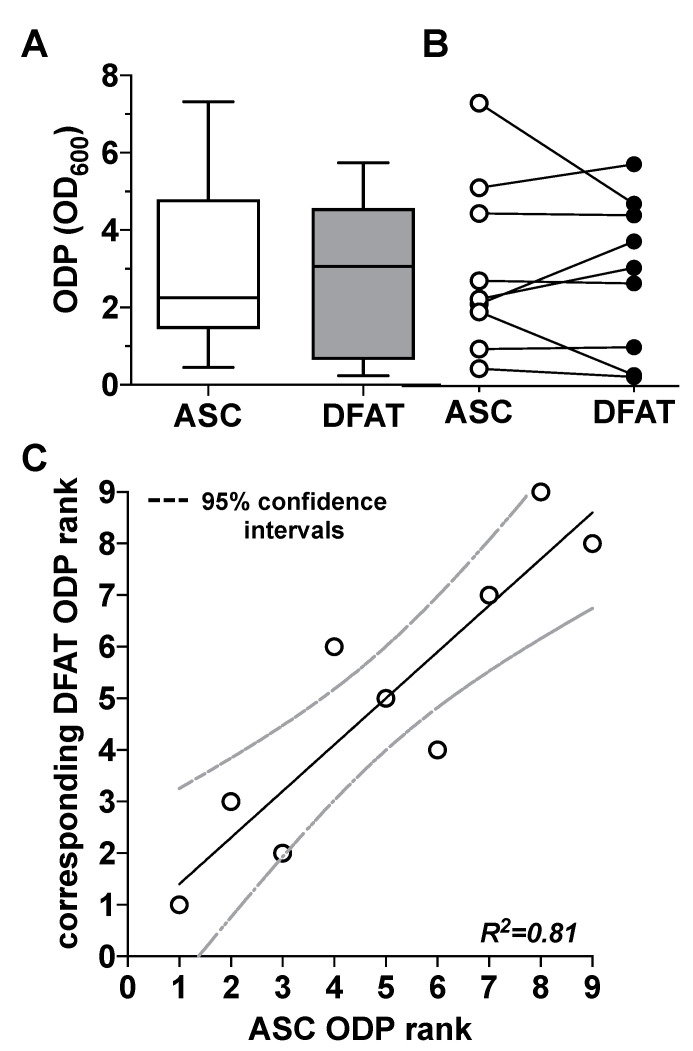
Comparison of the osteogenic differentiation potential (ODP) of ASC and DFAT cultures obtained from the same adipose tissue sample. (**A**) Quantitative evaluation of the osteogenic differentiation potential (ODP) of osteogenically activated ASC (white box) and DFAT cultures (grey box). The ODP values at day 14 of differentiation of 9 individual (*n* = 9) ASC or DFAT cultures are shown as boxplot with median value and with whiskers with minimum and maximum. As measurement parameter of the respective ODP value we used of the optical density of the Alizarin Red redissolution at 600 nm (OD_600_) of the cultures indicated. (**B**) Representation of the individual ODP values used in A of the ASC cultures examined (white circles) and of the corresponding DFAT cultures (black circles) which were produced from the same adipose tissue. (**C**) Rank analysis of the ODP values of the ASC cultures examined (X axis) and the corresponding ODP values of the DFAT cultures established from the same tissue (Y axis). The solid line represents the regression line (R^2^ = 0.81), the dashed lines indicate the 95% confidence intervals. *p* < 0.0005, slope deviation from zero.

When looking at the ODP values on day 14 of osteogenic differentiation, we were able to observe a very strong inter-individual range of variation in the ODP values of the ASC cultures used and the corresponding DFAT cultures. Thus, the highest ODP values of individual cell cultures were up to 20 times higher than the lowest values (Figure 3A). Further analysis of these data did not provide any evidence for a correlation of the said large inter-individual range of variation with the age of the adipose tissue donors. There was also no evidence of gender-specific factors that could explain this large inter-individual range of variation (Supplementary Figure S4). At the level of the individual ODP values of the individual cultures (Figure 3B), it became visible that ODP values of the ASC cultures correlated statistically significantly with the ODP values of the corresponding DFAT cultures. This is particularly evident from the results of a rank analysis shown in Figure 3C.

### 3.3. Impact of Oxidative Stress on Osteogenic Differentiation Potential of ASC and DFAT Cultures Obtained from the Same Adipose Tissue

**Figure 4:**
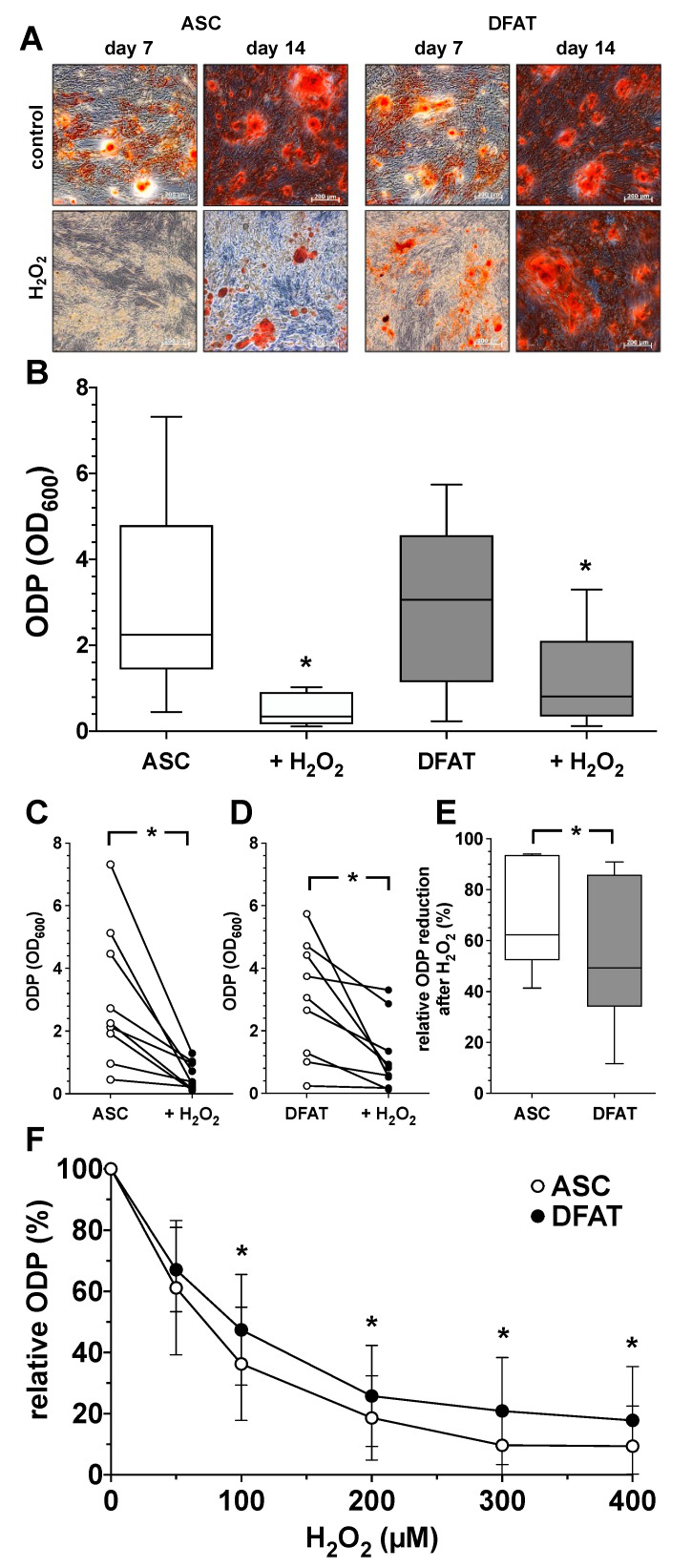
Impact of oxidative stress on osteogenic differentiation potential of ASC and DFAT cultures obtained from the same adipose tissue. (**A**) Alizarin Red staining of osteogenically activated ASC and DFAT cultures at day 7 and day 14 of differentiation (control) and respective cell cultures additionally maintained in the presence of 100 µM H_2_O_2_ (H_2_O_2_), scale bars 200 µM. (**B**) Quantitative evaluation of the osteogenic differentiation potential (ODP) of osteogenically activated ASC (white box) and DFAT cultures (grey box) and respective cell cultures additionally maintained in the presence of 100 µM H_2_O_2_. (+H_2_O_2_). The ODP values at day 14 of differentiation of 9 individual (*n* = 9) ASC or DFAT cultures are shown as boxplot with median value and with whiskers with minimum and maximum. As measurement parameter of the respective ODP value we used of the optical density of the Alizarin Red redissolution at 600 nm (OD_600_) of the cultures indicated. *, *p* < 0.05 as compared to the respective control culture maintained in the absence of H_2_O_2_. (**C**) Individual ODP values of the ASC cultures examined in (**B**) (ASC control cultures, white circles; H_2_O_2_-treated ASC cultures, black circles). (**D**) Individual ODP values of the DFAT cultures examined in (**B**) (DFAT control cultures, white circles; H_2_O_2_-treated DFAT cultures, black circles). (**E**) Relative decrease in ODP reduction at day 14 of differentiation after 14 day treatment with 100 µM H_2_O_2_. ASC (white box), DFAT cultures (grey box). (**C**–**E**), * *p* < 0.05. *n* = 9. (**F**) Change in the ODP of ASC (white circles) and DFAT cultures (black circles) detected at day 14 of differentiation after 14 day treatment with H_2_O_2_ at concentration indicated. * *p* < 0.05 as compared to the respective ASC cultures. *n* = 9.

In order to understand the influence of oxidative stress on the ODP of ASC and DFAT cultures, osteogenic differentiation was induced over a period of 14 days with the addition of 100 µM H_2_O_2_. As shown in Figure 4, the presence of the oxidative agent H_2_O_2_ led to a clear and concentration-dependent decrease in the ODP of ASC and DFAT cultures. After staining the extracellular matrix, an obvious reduction in the osteogenic process in H_2_O_2_-treated cultures was already apparent on day 7 of differentiation (Figure 4A). On day 14 this effect was even more noticeable. Morphologically, the differentiation-inhibiting effect of H_2_O_2_ was less prominent in DFATs than in ASC cultures (Figure 4A). Quantitative evaluation, showed that the oxidative stimulus induced a significant reduction in ODP in both cell types (Figure 4B–D) but the H_2_O_2_-induced reduction in osteogenic differentiation was significantly less pronounced in DFATs than in ASCs (Figure 4E,F).

### 3.4. Impact of Catalase on Osteogenic Differentiation Potential of ASC and DFAT Cultures Obtained from the Same Adipose Tissue

**Figure 5:**
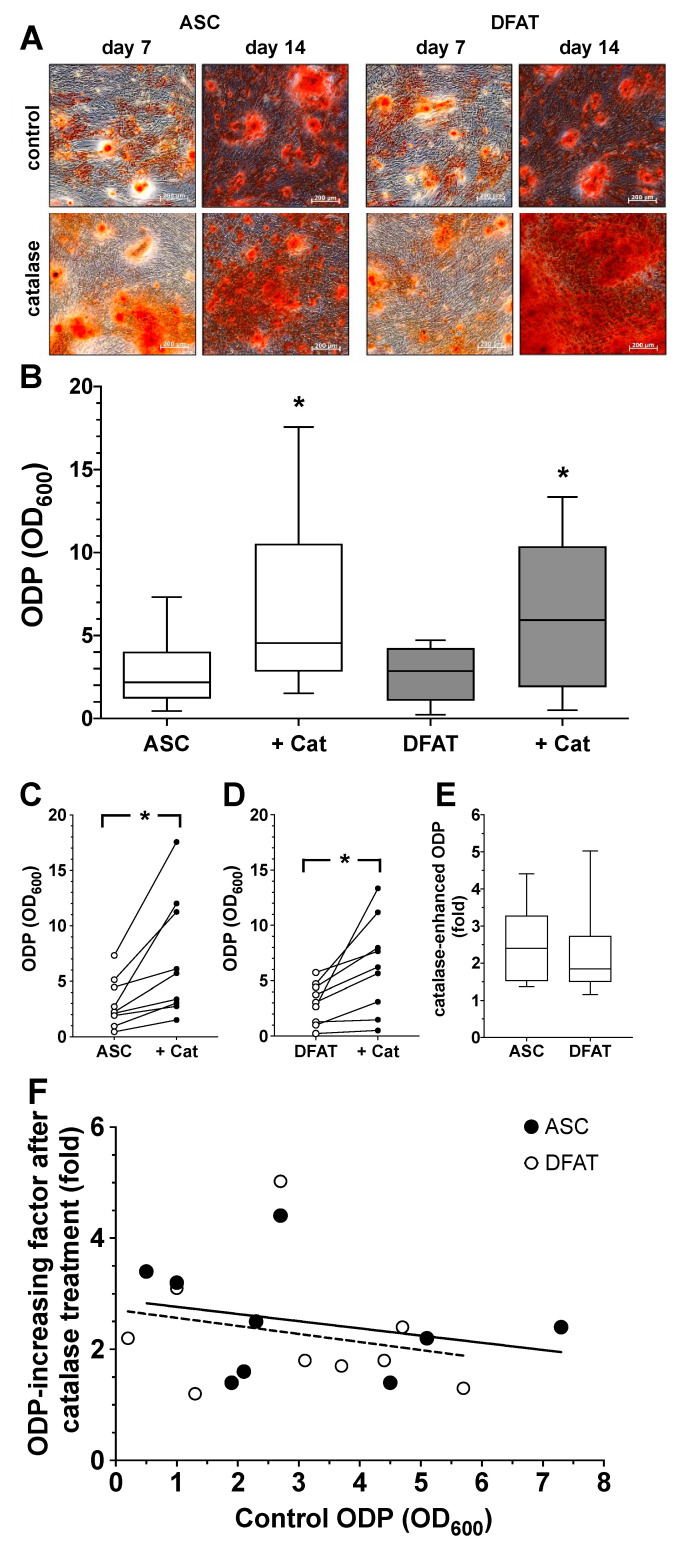
Impact of catalase on osteogenic differentiation potential of ASC and DFAT cultures obtained from the same adipose tissue. (**A**) Alizarin Red stain of osteogenically activated ASC and DFAT cultures at day 7 and day 14 of differentiation (control) and respective cell cultures additionally maintained in the presence of 250 U/mL catalase, scale bars 200 µM. (**B**) Quantitative evaluation of the osteogenic differentiation potential (ODP) of osteogenically activated ASC (white box) and DFAT cultures (grey box) and respective cell cultures additionally maintained in the presence of 250 U/mL catalase (+Cat). The ODP values at day 14 of differentiation of 9 individual (*n* = 9) ASC or DFAT cultures are shown as boxplot with median value and with whiskers with minimum and maximum. As measurement parameter of the respective ODP value we used of the optical density of the Alizarin Red redissolution at 600 nm (OD_600_) of the cultures indicated. *, *p* < 0.05 as compared to the respective control culture maintained in the absence of catalase. The control ASC and DFAT values shown here in Panel B are identical to the control ASC and DFAT values we show in Figure 4B.(**C**) Individual ODP values of the ASC cultures examined in (**B**) (ASC control cultures, white circles; catalase-treated ASC cultures, black circles). *p* < 0.05; *n* = 9. (**D**) Individual ODP values of the DFAT cultures examined in (**B**) (DFAT control cultures, white circles; catalase-treated DFAT cultures, black circles). *, *p* < 0.05; *n* = 9. (**E**) Relative increase in ODP (fold increase) detected at day 14 of differentiation after 14 day treatment with 250 U/mL catalase. ASC (white box), DFAT cultures (grey box). *n* = 9. (**F**) Correlation between ODP values (x-axis; control ODP) of the individual nine ASC (black circles) and DFAT cultures (white cultures) and the factor the ODPs were enhanced by addition of catalase (y-axis). The lines represent the regression lines obtained with ASC cultures (solid line; R^2^ = 0.07685) and DFAT cultures (dashed line, R^2^ = 0.05155), respectively. *p* < 0.0005, slope deviation from zero.

**Figure 6:**
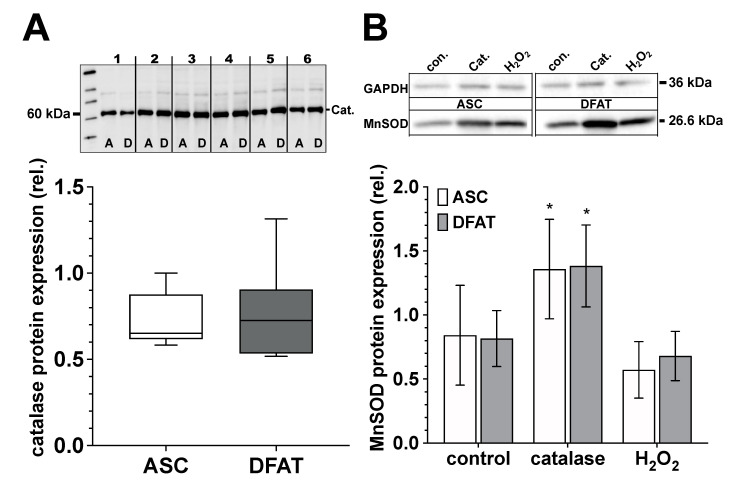
Catalase and MnSOD protein expression of ASC and DFAT cultures obtained from the same adipose tissue. (**A**) Relative catalase protein expression in ASC (white box) and DFAT cultures (gray box) of the same adipose tissue origin. Values are shown as boxplot with median value and with whiskers with minimum and maximum. The specific signal of the catalase expression was normalized to the signal of the protein content of the whole corresponding lane. The representative photograph of the blot shows the protein expression of catalase in ASC (**A**) and DFAT cultures (**B**), which were established from six individual adipose tissue samples. (**B**) Quantification of MnSOD protein expression in control, catalase-treated (250 U/mL) and H_2_O_2_-treated (100 µM) ASC (white bars) and DFAT cultures (gray bars) of the same origin of adipose tissue. The bars show the mean ± SD of four individual experiments. *, *p* < 0.05 as compared to the controls. The specific MnSOD signals were normalized in comparison to the corresponding GAPDH in the individual sample. The representative photograph of the blot shows the protein expression of MnSOD and GAPDH in ASC (**A**) and DFAT cultures (**B**) after the cultures have been treated accordingly.

Based on the experiment described above, we also evaluated the effect of the enzyme catalase, a relevant factor in the cells defense against oxidative stress, on the ODP of ASC and DFAT cultures. We could already see at day 7 of differentiation that catalase visibly increased osteogenesis in both cell types (Figure 5A). The quantification of the results confirmed this observation. Catalase led to a significant increase in ODP in ASCs as well as in DFATs (Figure 5B–D). When looking at the individual values of the catalase-induced ODP of both cell types, it could be seen that the relative increase was very similar regardless of the starting value of the controls. The data from the individual values summarized in Figure 6E make it clear that the mean values of the catalase-elevated ODP were relatively constant, approximately 2- to 2.5-fold higher than the initial values, regardless of how large or small the original individual ODP of the respective cell cultures was (Figure 5F).

### 3.5. Catalase and MnSOD Protein Expression of ASC and DFAT Cultures Obtained from the Same Adipose Tissue

ASC and DFAT cultures of the same adipose tissue origin show no significant differences in terms of their catalase and MnSOD protein expression (Figure 6). Regarding MnSOD, both cell types show a significant increase in protein expression after treatment with catalase (250 U/mL) and after treatment with H_2_O_2_ (100 µM) we did not observe any significant effect on MnSOD protein expression.

## 4. Discussion

Due to the constantly growing development of novel regenerative therapies, there is also an increasing need for suitable sources for MSCs. Human adipose tissue abundant in ASCs represents a very attractive source for cell therapeutic measures [45]. ASCs are relatively easy to isolate and have a low risk of side effects and health complications. A possible disadvantage in the therapeutic use of ASCs is the large cellular heterogeneity of this cell fraction. In addition to preadipocytes, the stromal fraction of adipose tissue also contains fibroblasts, endothelial cells and their precursor variants and possibly other cell types. In addition, the cells of the adipose tissue capable of differentiation occur in variable proportions in the obtained stromal fraction and also have various differentiation potentials, e.g., in regard to their osteogenic capacity. From our own experience as shown here, but also from numerous publications, we know that due to their intrinsic heterogeneity the tissue-specific differentiation potential of an individual ASC preparation cannot be reliably predicted [46,47,48]. It seems that a more uniform cell population with a multilineage differentiation potential would allow for a better and more reliable differentiation outcome. 

One aim of the current study was to evaluate this assumption. To this end, we compared the osteogenic differentiation potential of ASC cultures with that of dedifferentiated adipocytes (DFAT) of the same adipose tissue sample origin. DFATs derived from mature adipocytes, unlike heterogenic ASCs represent a highly homogeneous cell population. Still DFATs show a nearly identical antigen phenotype like ASC cultures. Furthermore, they were able to grow adherently and, like ASCs, could be effectively differentiated into adipogenic, chondrogenic and osteogenic lineage. In a more differentiated analysis of the osteogenic differentiation potential (ODP), we were able to observe that the ODP of a DFAT culture correlated significantly with the ODP of the corresponding ASCs from the same donor. Thus, a donors ASC with low ODP had also a corresponding DFAT with a low ODP and vice versa. In the examined tissue samples the correlation of the ODP of the generated ASCs and the corresponding DFATs was highly significant. Therefore, it seems that the ODP of both was not primarily a function of the cell type or culture conditions but moreover appears to be a function of the adipose tissue from which the cultures were established. These very important results, contradict earlier findings, where a fundamentally higher ODP could be observed in the DFAT cultures compared to ASC cultures [49,50]. However, another study focusing ASCs and DFATs of 12 elderly osteoporotic donors confirmed the correlation of ODP quality observed by us [51]. 

Regardless of the clear correlation just discussed, we observed an enormous interindividual variation in the ODP of the isolated ASC and DFAT cultures, which in our case showed differences by a factor of up to 20. With such a large range of interindividual ODPs, it is almost impossible to define a threshold value or fixed point that distinguishes a “normal” from an unphysiological or even “pathophysiological” low ODP. Donor variation is a common issue in osteogenic differentiation of MSCs, and has been described by others for instance for BMSCs [47,48] but also for human ASC cultures [52]. What can be the molecular mechanism for such a strong interindividual fluctuation? 

It has been shown, that obesity has a huge impact on ASCs osteogenic performance. Cells of obese donors drastically failed to accumulate calcium, indicated by Alizarin red staining, and also expressed less osteogenic proteins like RUNX2, ALP and osteocalcin compared to lean control groups [6,19,53]. Due to the largely anonymized transfer of the test material, we unfortunately have no information on the body mass index (BMI) of our donors. About 30% of the tissue material we used came entirely from aesthetically motivated liposuctions. Here, we can almost certainly assume that obesity patients were not recorded. However, 70% of the adipose tissue samples came from abdominoplasty samples that were obtained as part of surgical measures to tackle pre-existing obesity. Even if the absolute number of donors of 9 does not allow any reliable statistical statements, we did not observe any involvement of obesity-relevant factors that might give an explanation for the large fluctuation range of ODPs that we observed. 

In addition, there was no obvious pattern between the age of donors and the wide spread of ODP values. In the past, some authors have found a clear correlation between increasing age of donors and a decrease in ODP of MSCs [54,55]. Like other authors before us, we could not find any statistically significant indications of age-related differences or an influence of gender on the osteogenic differentiation potential, which we recorded in Figure S5 in the Supplementary Materials. Nevertheless, a tendency towards higher inter-individual variation in ODP with increasing age could be observed by some [56].

A generally accepted view is that aging processes and senescence correlate with an increase in cellular oxidative stress [57,58]. This oxidative stress is also associated with the development of various degenerative and metabolic diseases [59,60,61]. Oxidative stress is also seen as a negative pathogenic factor in connection with osteoporosis [22,62,63,64,65] and can significantly inhibit stem cell osteogenesis in vitro as well as in vivo [20,22]. Therefore, in analogy to the comparison of the ODP of both ASC and DFAT, we also compared the influence of oxidative stress on the osteogenic behavior of both. To simulate oxidative stress, we used H_2_O_2_, a physiologically relevant reactive oxygen species (ROS) [60] that is often used as a prooxidant. In this experimental setup it became clear that DFAT cultures were significantly more resistant than ASC cultures in regard to the damage caused by oxidative impact. In the concentration range of the used H_2_O_2_, which was not directly toxic to the cells (100 µM), the oxidative stimulus caused a significant decrease in ODP in cultures of both cell types. However, this decrease was significantly less pronounced in DFATs than in ASCs. This finding suggests that DFATs‘ enzymes may have better antioxidant performance and therefore cells have higher antioxidant capacity. This was ruled out for catalase and MnSOD, since we could not observe any differences in their protein expression between the two cell types. We therefore believe that the molecular mechanism counteracting oxidative stress-suppressed osteogenic differentiation capacity in osteogenically activated DFAT cultures is not mediated by catalase or MnSOD, but by other potent antioxidant or hydrogen peroxide depleting systems of the cells, such as glutathione peroxidase/glutathione system or the glutathione/thioredoxin/peroxiredoxin system [66,67].

Based on the assumption that very low individual ODP might be due to increased intracellular ROS exposure, an antioxidant intervention should lead to improved and increased ODP values. In cell cultures with high ODP values, the influence of the antioxidant measures should be correspondingly lower. Our experimentally collected data only partially met these expectations. In cultures of both cell types, which we internally call osteogenic “low-responder” cultures due to the very low achievable ODP values, an antioxidant treatment with exogenously added catalase led to a significant increase in ODP by a mean factor of about 2.5. However, the factor of the catalase-induced increase in osteogenesis was approximately the same in all cultures, regardless of individual ODP of the respective culture without catalase. This means that the ODPs of low responding as well as the ODPs of highly (or assumed maximal) responding cells were increased by the same factor by catalase. These findings suggest that the strong interindividual variation of the observed ODP is idiopathic, but appears to neither depend on the age or BMI of donors nor does it seem to correlate with increased oxidative stress. Thus, the critical question remains unanswered as to whether the very low ODP of some individuals observed here in vitro would be a clue to an underlying disease in vivo.

Regardless of the (patho)-physiological question of a very low ODP in vivo, it seems to be undisputed that in most bone regenerative therapy approaches, including tissue engineering of bone tissue, rapid and strong osteogenesis would have an enormous advantage and the use of exogenous added catalase could be an effective tool. However, how could one imagine the molecular mechanism of the effect of catalase observed here? H_2_O_2_ is a rather complicated cell signaling factor that acts as a second messenger. The signaling mechanism is based on a continuous flow of H_2_O_2_ between the cytoplasm and mitochondria, but also between the cytoplasm and the extracellular space, due to the concentration gradient. The increase in the H_2_O_2_ concentration in one compartment leads to a concentration gradient and consequently to a modified H_2_O_2_ flow into the other compartments. Here, mitochondria serve as reservoir areas for H_2_O_2_. An increase in the local H_2_O_2_ concentration as a result of adding the substance to the cell medium therefore not only leads to an increase in the H_2_O_2_ concentration in the cytoplasm, but also to a correspondingly graduated increase in the mitochondria [68]. Conversely, the extracellular application of catalase to the cell culture media has a significant impact on the H_2_O_2_ balance between the extracellular space, cytoplasm and mitochondria. Due to the catalase-related depletion of extracellular H_2_O_2_, diffusion of H_2_O_2_ out of the cell via the cell membrane or aquaporins [69] follows the concentration gradient. As a result of these events, there is a further diffusion-related flow of H_2_O_2_ from the mitochondria in the direction of the cytoplasm and thus the H_2_O_2_ effects in the mitochondria are reduced or blocked. An indicator for this is the raised MnSOD expression in catalase treated cells, probably in an attempt to restore mitochondrial H_2_O_2_. How other SODs might be affected needs to be investigated. These assumptions are based on the “steady-state H_2_O_2_ concentration” model proposed by Starkov and Treberg [68,70]. In this model, it is postulated that mitochondria have the ability to absorb excess H_2_O_2_ in the sense of a reservoir and that this excess H_2_O_2_ can be made available to the cell again even in the case of unphysiologically low extramitochondrial H_2_O_2_ concentrations [64,66]. However, it is not possible to say at this point whether depletion of H_2_O_2_ by catalase in the cytoplasm or mitochondria is the mechanism leading to increased osteogenesis, or whether catalase-induced disruption of intracellular H_2_O_2_ signaling pathways is the key factor.

In conclusion, we found that ASCs and DFATs of the same adipose tissue had very similar osteogenic capacity. Furthermore, variation in ODPs between donors was very apparent. Even with the underlying mechanism unknown, we want to highlight catalase as a beneficial addition to osteogenic induction medium or osteogenic biomaterial scaffolds for the tissue engineering of bone tissue. Catalase was able to improve osteogenesis about 2–2.5 fold in ASCs and DFATs in all donors despite donor variation. 

## Data Availability

The datasets used and/or analyzed during the current study are available from the corresponding author on reasonable request.

## Supplementary Materials

The following supporting information can be downloaded at: https://www.mdpi.com/article/10.3390/biomedicines10123071/s1, Figure S1: Generation of DFAT by ceiling culture; Figure S2: Exemplary histogram plots for cell-surface antigens; Figure S3: Correlation between the osteogenic differentiation potential of ASC and DFAT cultures; Table S1: Table of donor data.
