# Supplementary material for: Comparative Study of the Osteogenic Differentiation Potential of Adipose Tissue-Derived Stromal Cells and Dedifferentiated Adipose Cells of the Same Tissue Origin under Pro and Antioxidant Conditions
Source: Biomedicines. 2022 Nov 29;10(12):3071. doi: 10.3390/biomedicines10123071 (PMC9776284; doi:10.3390/biomedicines10123071)
Supplement: Supplementary file 1 [file biomedicines-10-03071-s001.zip › biomedicines-1957856-supplementary.pdf]

biomedicines

MDPI

Table S1. Table of donor data.

donor	osteogenic response	gender	age	type of surgery
1	medium	female	35	liposuction
2	high	unknown	unknown	liposuction
3	medium	male	60	abdominoplasty
4	low	female	unknown	abdominoplasty
5	low	female	48	liposuction
6	high	female	33	abdominoplasty
7	medium	male	61	abdominoplasty
8	low	female	20	abdominoplasty
9	high	female	70	abdominoplasty

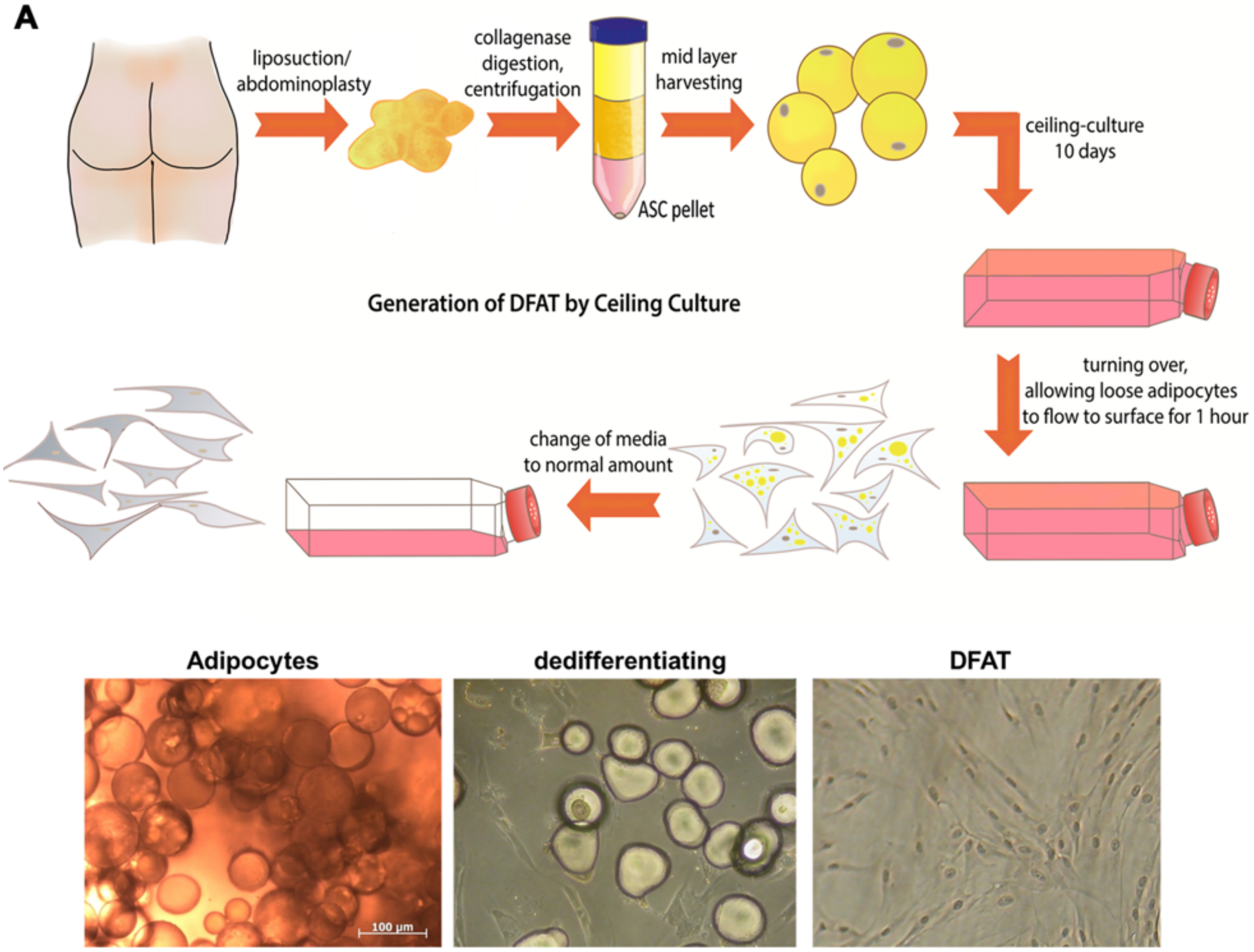

Supplementary Figure S1. Generation of DFAT by ceiling culture. After isolation of fatty tissue by liposuction or abdominoplasty, minced tissue was digested with collagenase and after centrifugation the middle layer, consisting of mature adipocytes was transferred into cell tissue flasks and filled up to the brim with medium. Flasks were cultured bottom up for 10 days, in which adipocytes stick to the top (bottom) of the flask and start dedifferentiating. After 10 days flasks were turned and after one hour medium was changed to normal amount. After 1-3 days DFATs were harvested and seeded to larger flasks for further expansion. Photos show mature adipocytes dedifferentiating in about 10 days of ceiling culture and after passage showing a uniform cell type with a fibroblast-like phenotype.

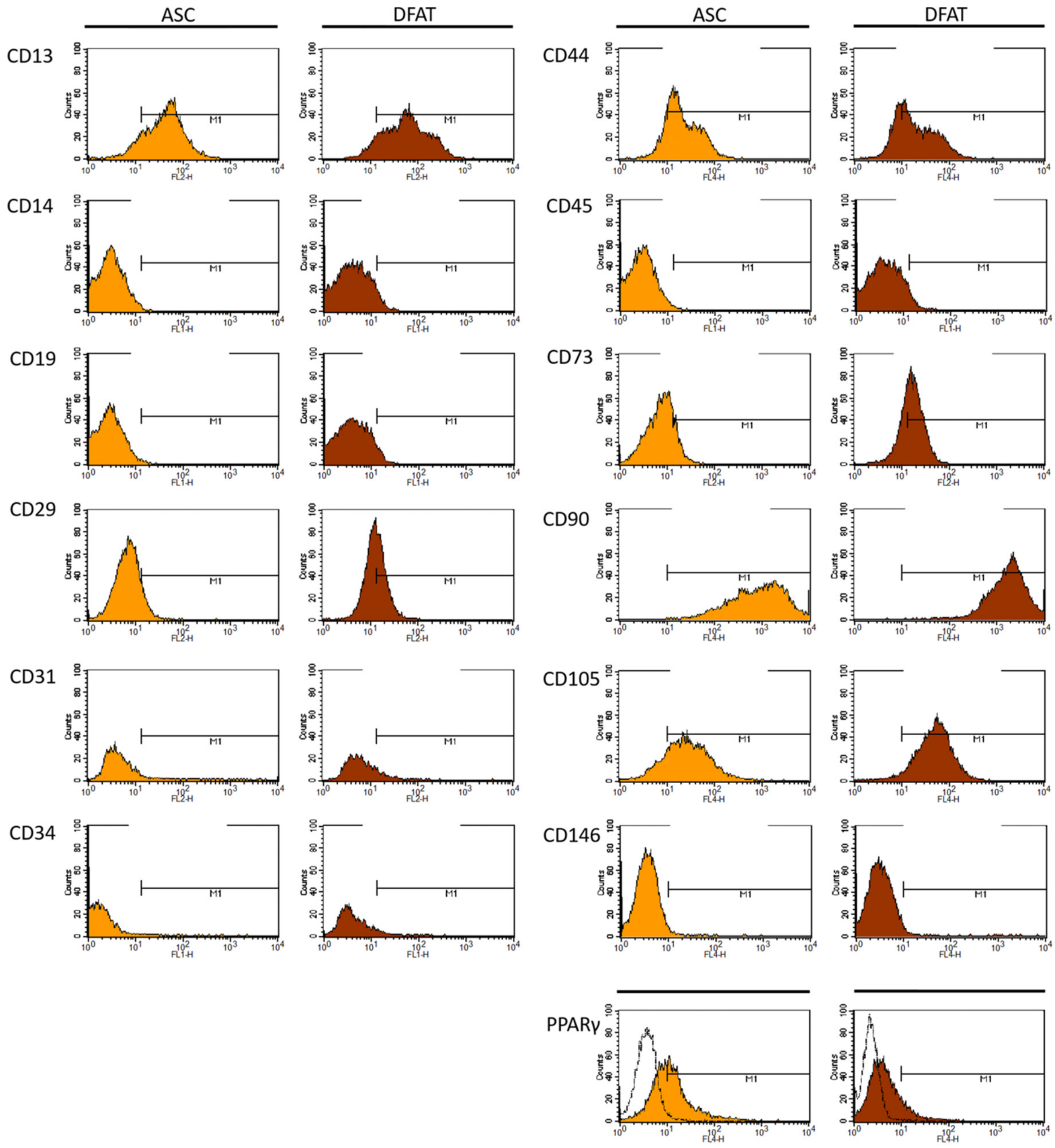

Supplementary Figure S2. Exemplary histogram plots for cell-surface antigens, analyzed by flow cytometry, of a typical donors ASC (orange) and corresponding DFAT (red). PPARγ was stained after permeabilisation of the cells. Black curve outline indicates 2nd antibody only analysis.

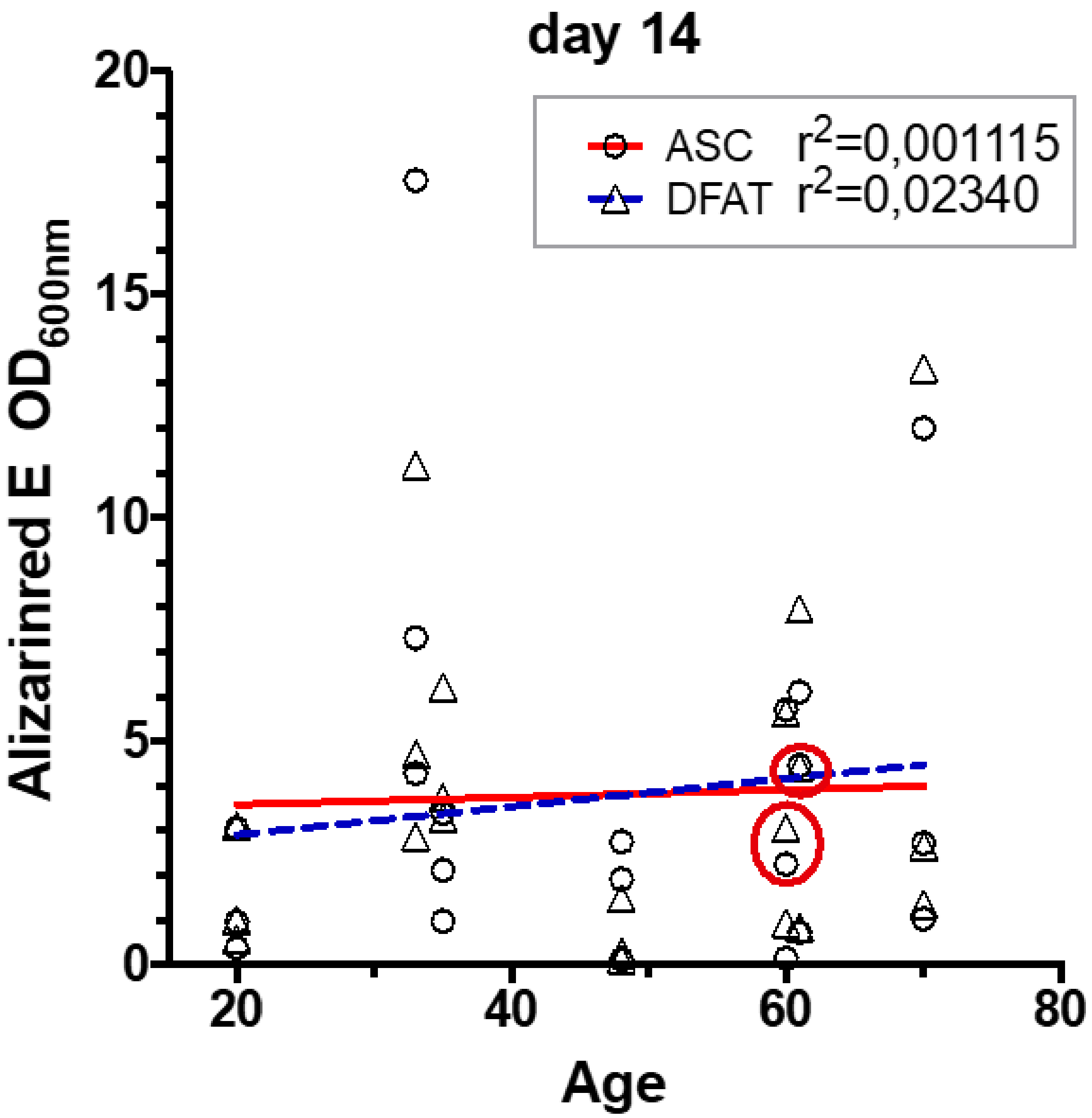

Supplementary Figure S3. Correlation between the osteogenic differentiation potential of ASC (open circles) and DFAT cultures (open triangles) of identical adipose tissue origin and the age of the adipose tissue donors. The red line represents the linear regression of the data collected with the ASC cultures and the dashed blue line that of the DFAT cultures. In both cases the slope is not significantly different from zero. The values surrounded by red circles represent the ODM values of the two male adipose tissue donors included in the experiment.
